# Editorial: Bridging Science and Policy for Surveillance, Economics and Social Sciences: ICAHS and ISESSAH 2020

**DOI:** 10.3389/fvets.2021.790248

**Published:** 2021-11-18

**Authors:** Lis Alban, Carola Sauter-Louis, Victoria J. Brookes, Chris J. M. Bartels, Bouda Vosough Ahmadi, Jonathan Rushton, Salome Dürr

**Affiliations:** ^1^Danish Agriculture and Food Council, Copenhagen, Denmark; ^2^Department of Veterinary and Animal Sciences, University of Copenhagen, Copenhagen, Denmark; ^3^Institute of Epidemiology, Friedrich-Loeffler-Institute, Greifswald, Germany; ^4^Sydney School of Veterinary Science, University of Sydney, Camperdown, NSW, Australia; ^5^Food and Agricultural Organization, Ulaanbaatar, Mongolia; ^6^The European Commission for the Control of Foot and Mouth Disease (EuFMD), Animal Production and Health Division (NSA), Food and Agricultural Organization, Rome, Italy; ^7^Veterinary and Ecological Sciences, Faculty of Health and Life Sciences, Institute of Infection, University of Liverpool, Liverpool, United Kingdom; ^8^Veterinary Public Health Institute, Vetsuisse Faculty, University of Bern, Bern, Switzerland

**Keywords:** animal health, zoonoses, food safety, epidemiology, economics, surveillance

African swine fever, antimicrobial resistance and the release of zoonotic pathogens from food systems are examples of three global challenges in animal health, which are currently threatening the world. These hazards are associated with significant economic, societal and food security issues for a growing number of countries. The movement of people, animals and food has increased to a point where major disease events are occurring with increased frequency, challenging our ability to manage these problems in a timely and proportionate manner. Until better animal health surveillance and associated response measures are adequately resourced, the challenge society faces will continue to grow. The COVID-19 pandemic indicates that simply acting on emergence and spread is not appropriate. Preparedness is warranted to prevent, detect and control outbreaks before they become large.

In the process of preventing and mitigating the risks and impacts of these challenges, surveillance is a key element. It enables an understanding of the actions needed - for example, why and where actions are required. The social, economic and cultural context needs to be understood for the implemented mitigating actions to have the greatest probability of success in the prevention and control of animal and zoonotic threats.

Available solutions are dynamic because they are influenced by the societies affected and their associated side-effects, such as constraints on trade and food security. Moreover, what is considered feasible in one country might not be feasible in another, or it might be considered not worth doing by decision-makers because other surveillance options or challenges are perceived as more important. Learning from and sharing each other's experiences are therefore pivotal for successful control and mitigation of cross-border challenges. Hence, the actors involved in surveillance and control - whether they are affiliated with government authorities, academia or livestock industries - need to exchange views and experience to be able to collaborate effectively in a transdisciplinary way. In the future, this may result in the development of new ways of collaboration using cross-sectoral and interdisciplinary approaches such as through Public-Private-Partnerships. Much can probably be obtained through such alternative governance models - if people know how to do it.

The International Conference on Animal Health Surveillance (ICAHS) and The International Society for Economics and Social Sciences of Animal Health (ISESSAH) provide an opportunity for learning and sharing between academic researchers, representatives of the food supply chain, authorities, as well as people working for international organizations within food safety and food security, and animal health from all over the world. Although the joint conference planned in May 2020 was canceled due to the COVID-19 pandemic, the authors of research which were selected to be part of the joint ICAHS4/ISESSAH conference were invited to submit their work to this Research Topic. The areas covered include the following topics, identified by the international Scientific Committee for the ICAHS4 and ISESSAH conference:

- Integrating novel methods in surveillance
- Use of surveillance data
- Cross-sector surveillance – organization, collaboration and benefits
- Translating surveillance outcomes into policy, decisions and actions
- Costs and motivation
- Social science in the control of animal diseases
- Economic considerations in animal health

The Research Topic consists of 16 papers of which three were brief research reports and 13 original research articles. The papers report work undertaken in Africa, Australia, Europe, Southeast Asia, as well as South- and North America. Fish, chickens, pigs, sheep, bovines, horses as well as ungulates as a group were the livestock species studied. The specific hazards were Classical and African Swine Fever, Foot-and-Mouth Disease, *Salmonella*, Psoroptes ovis causing sheep scab, antimicrobial use (AMU) and resistance (AMR), *Vibrio* as well as One Health and zoonotic infections in general.

Regarding integrating novel methods in surveillance, Sandberg et al. report from an ongoing scientific network project called CoEvalAMR dealing with how to assess evaluation tools for AMU and AMR. The authors conclude that there are many tools available which each have their advantages and disadvantages, making it pertinent to choose a method which fits the objective of evaluation.

Three papers describe the use of surveillance data. The first by Desvaux et al., reports an analysis of the effect of strengthened surveillance to support African Swine Fever prevention in France at the border of Belgium during the outbreak of African Swine Fever in Belgium. The objective of the strengthened surveillance was to assure early detection and to support the free status of the zone. Tuat et al. report from a pilot surveillance programme for AMR in pigs and chickens in Vietnam, enabling them to map the prevalence of different kinds of AMR. The authors conclude that establishment of an annual surveillance programme for AMR in livestock is needed in Vietnam. Finally, Veldhuis et al. investigated the added value of meat inspection data for monitoring of dairy cattle health in the Netherlands. Seven indicators were judged to add value to the existing cattle health surveillance components, as they provided either new information or information regarding specific health problems.

Two papers describe cross-sector surveillance–organization, collaboration and benefits. The first is by Thomas et al., who studied the cross-sectoral zoonotic disease surveillance in place in Western Kenya using interviews with 28 disease surveillance officers from the human and animal health sectors. The study points to the challenges related to the lack of formal operational structures and poor allocation of resources. Schettino et al. have undertaken a risk assessment regarding the introduction of Classical Swine Fever into Mato Grosso in Brazil. The authors identified two major pathways; the first dealt with shipment of commercial pigs and the other with movement of wild boars. The conclusion was that the strategies for surveillance must target the specific route of entry.

Translation of surveillance outcomes into policy, decisions and actions is covered by three papers. The first of these is by Geddes et al., who investigated how scanning surveillance can be used to inform future strategies for the control of endemic diseases, using sheep scab as an example. The work undertaken led to an enhancement of the knowledge of sheep scab, identified areas for targeted action, and offered a framework for assessment of impact of disease control initiatives. The second paper is by Capon et al., who in a simulation study assessed the use of vaccination against Foot-and-Mouth Disease outbreaks across Australia. Several scenarios were investigated. The conclusion was that selective, targeted vaccination strategies could achieve effective control, while significantly reducing the number of animals vaccinated. The third paper is by Dórea and Revie, who reviewed the opportunities for connecting data and generating information to support decision-making. The authors focus on the challenges related to the increasingly complex dimensions of data in population health, and how to enable data-driven surveillance to go beyond signal detection and support an expanded set of surveillance goals.

Two papers deal with costs and motivation. The first is by Olsen et al., who studied Danish pig farmers' perceptions of the existing economic incentives to control *Salmonella* prevalence at herd level. The results support the idea of an outcome-based *Salmonella* penalty scheme that is presently in place. However, the large uncertainties about costs and effects toward *Salmonella* control might hamper the effectiveness of the penalty system as a regulatory instrument to influence farmer behavior. The second paper is by Urner et al., who investigated the perceptions of Estonian and Latvian hunters regarding the control of African Swine Fever. There were mainly similarities in hunters' perceptions between the two countries, although the passive surveillance in Latvia was perceived more as an ethical duty than driven by incentives. The results highlight further opportunities for improving the cooperation with hunters in the future.

Aspects related to social science in the control of animal diseases are covered in three papers. The first of these is by Pudenz et al., who studied US cattle producers' adoption of the Secure Beef Supply Plan, which is focusing on enhancing biosecurity practices and preparedness for Foot-and-Mouth Disease. The authors found that the adoption of the pre-outbreak practices is likely to be low because the benefits of adopting the practices depend on an event, which is associated with a low and uncertain probability. Özçelik et al. investigated the potential and challenges of community-based surveillance in animal health, using a pilot study among equine owners in Switzerland. The ambition was to assess the use of community members other than health care professionals for reporting health events. One conclusion is that it is questionable whether the added value of the generated surveillance balances the efforts necessary to implement a successful system. Finally, Bordier et al. studied how to engage stakeholders in the design of One Health surveillance systems through a participatory approach. The study was undertaken in Vietnam and in France. It identified that the engagement of the stakeholders in a participatory process must be sustained to ensure the implementation of co-constructed solutions and to evaluate their effectiveness and impacts.

Economic considerations in animal health are covered in two papers. Yazid et al. estimated the economic loss due to vibriosis in net-cage cultured Asian seabass. The case was based on evidence from the East coast of the Malaysian peninsular. Asian seabass production has contributed substantially to Malaysia's economic activities and food security. It is concluded that more focus is needed regarding control and prevention of vibriosis infection from the hatcheries. Vredenberg et al. made an empirical analysis of the longevity of dairy cows in relation to economic herd performance, using data from the Netherlands. The results show that the gross margin was not significantly associated with the age of the culled cows or lifetime milk production of culled cows. Moreover, the authors conclude that this implies that there is a potential for increasing longevity to meet society's concerns on animal welfare and environmental pollution without affecting the economic performance of the herd.

## Author Contributions

LA took the lead in writing the Editorial and received comments from the co-authors. All authors of the editorial contributed as editors to the production of the research theme.

## Conflict of Interest

LA works for an organization that gives advice to the Danish farmers and meat industry. The remaining authors declare that the research was conducted in the absence of any commercial or financial relationships that could be construed as a potential conflict of interest.

## Publisher's Note

All claims expressed in this article are solely those of the authors and do not necessarily represent those of their affiliated organizations, or those of the publisher, the editors and the reviewers. Any product that may be evaluated in this article, or claim that may be made by its manufacturer, is not guaranteed or endorsed by the publisher.

